# Menstrual Pain Management, School Absenteeism and Educational Performance Among Adolescent Students: Cross‐Sectional Mixed‐Methods Analysis Nested Within a Cluster Randomised Trial

**DOI:** 10.1111/1471-0528.70094

**Published:** 2025-11-24

**Authors:** Prossy Namirembe, Alice Nassanga, Christopher Baleke, Beatrice Nanyonga, Ronald Kyasanku, Sophie Belfield, Denis Ssenyondwa, Esther Martha Haruri, Shamirah Nakalema, Katherine A. Thomas, Denis Ndekezi, Kate Andrews Nelson, Belen Torondel‐Lopez, Helen A. Weiss

**Affiliations:** ^1^ MRC/UVRI and LSHTM Uganda Research Unit Entebbe Uganda; ^2^ WoMena Uganda Kampala Uganda; ^3^ Cornell College of Human Ecology Cornell University Ithaca New York USA; ^4^ International Statistics and Epidemiology Group London School of Hygiene & Tropical Medicine London UK; ^5^ Faculty of Infectious and Tropical Diseases London School of Hygiene and Tropical Medicine London UK

**Keywords:** adolescent health, menstrual pain, pain management, school absenteeism

## Abstract

**Objective:**

To describe menstrual pain and pain management, and the associations of pain relief with school absenteeism and educational performance, among Ugandan adolescents.

**Design:**

Cross‐sectional mixed‐methods study, nested within a school‐based cluster randomised controlled trial.

**Setting and Population:**

Adolescent girls in 60 secondary schools in Uganda.

**Methods:**

Quantitative surveys, focus group discussions and in‐depth interviews were conducted. Data were analysed using thematic framework analysis for qualitative data, and random‐effects regression analysis for quantitative data.

**Main Outcome Measures:**

Self‐reported pain during the last menstrual period (LMP), school absenteeism and educational performance.

**Results:**

Among 2683 participants, the majority (*n* = 2227; 83.0%) reported pain during their LMP and were more likely to miss school than those without pain (mean days missed per month 2.1 vs. 1.4; adjusted incidence rate ratio [aIRR] = 1.46, 95% CI 1.30–1.65). Participants reported that menstrual pain hindered their ability to engage at school, leading to reduced class attendance and participation. Pain management strategies (painkillers, warm water bottles, stretching, or exercise) were reported by 1587 (71.3%) participants with pain at LMP, and were less commonly reported among participants with mental health problems and poorer menstrual self‐efficacy. Participants who did not report pain relief missed more school days than those who did report pain relief (aIRR = 1.39, 95% CI 1.11–1.74).

**Conclusion:**

Menstrual pain without effective relief was associated with increased school absenteeism. There is a need to improve the uptake of effective pain relief strategies in Ugandan schools and similar settings.

## 1 Introduction

Dysmenorrhea (menstrual pain) is prevalent among females, especially adolescents and young women [1]. A systematic review among women aged < 25 years found a mean prevalence of 71.1% (95% CI 66.6%–75.2%), similar in 15 high‐income and 23 low‐middle income countries [1].

Primary dysmenorrhea (PD) is menstrual pain with no underlying pathology and is the most common type of dysmenorrhea in adolescents [2]. Secondary dysmenorrhea has an identifiable cause, most commonly endometriosis [3]. PD severity has been associated with younger age, lower parity, non‐use of oral contraceptives, family history of dysmenorrhea and stress [4]. Dysmenorrhea can impact educational outcomes, social participation, physical activity, mental health and sleep [5]. In the systematic review, 20.1% (95% CI 14.9%–26.7%) of participants reported absence from school or university due to dysmenorrhea (with significant heterogeneity), and 40.9% (95% CI 28.3%–54.9%) reported adverse effects on classroom performance or concentration [1]. Another systematic review, among university students globally, found that students with severe pain had more absenteeism and reduced engagement than those with no or less pain [6].

There is little evidence on the impact of pain management strategies on menstrual‐related absenteeism or educational performance [1, 6]. The recommended first‐line treatments for dysmenorrhea are non‐steroidal anti‐inflammatory drugs (NSAIDs) [7] or hormonal contraception [8]. Globally, about half of young women report using painkillers (48%), and few report using hormonal contraceptives especially in low‐ and middle‐income countries (LMICs) (< 1%) [9]. Non‐pharmacological interventions including application of heat, stretching, and rest [10] may play an important role, but there is little evidence on their efficacy [11].

Our previous research in Ugandan secondary schools showed that dysmenorrhea is common (74.3%, 95% CI 68.1%–80.0%) [12], and impacts education, mental health and quality of life [13]. Prevalence was similarly high among female undergraduates (63.6%–75.8%) [14, 15]. However, studies from Uganda and Tanzania show limited access or acceptability of painkillers and oral contraceptives for menstrual pain management among in‐school adolescents [16, 17]. A cluster‐randomised trial evaluating the impact of a multi‐component menstrual health intervention in Ugandan secondary schools on menstrual health, mental health and educational outcomes showed a modest impact of the intervention on use of effective pain management strategies at last menstrual period (LMP) (75.4% vs. 66.6%; adjusted odds ratio [aOR] = 1.50, 95% CI 1.25–1.80) [18].

The aim of this paper is to assess whether effective management of pain during menstruation is associated with reduced school absenteeism and educational performance in Uganda. The objectives are to (i) describe pain during menstruation and pain management experiences among Ugandan secondary students; (ii) assess factors associated with menstrual pain management and pain relief; and (iii) estimate associations of pain management with school absenteeism and educational performance.

## 2 Methods

### 2.1 Study Design and Setting

A cross‐sectional mixed‐methods analysis was conducted nested within a school‐based cluster randomised controlled trial (MENISCUS) of the impact of a menstrual health intervention on health and educational outcomes among secondary school girls in Uganda. The intervention effects are published [18]. Schools were eligible if they were in Wakiso or Kalungu districts, mixed‐gender, had both day and boarding students, and basic water, sanitation and hygiene (WASH) facilities [19]. We obtained written school‐level consent from the headteacher or a representative in a random sample of 60 eligible schools.

The intervention comprised training teachers to improve puberty education, a student‐led drama skit about menstrual health, training school members to deliver menstrual health education sessions alongside the distribution of kits containing reusable menstrual products, provision and training in pain management strategies, and improved school WASH facilities [19]. The pain management component included (i) education on managing menstrual pain (understanding menstrual pain, exercise, stretching, warm water bottles and analgesic use), (ii) information on common misconceptions around pain management (e.g., that painkillers cause infertility); and (iii) vouchers for redeeming upto 6 painkiller tablets per month (paracetamol 500 mg or ibuprofen 200 mg) from a trained school nurse or teacher.

### 2.2 Study Population and Data Collection

#### 2.2.1 Quantitative Data

We collected survey data at baseline (21st March to 5th July 2022) and endline (5th June to 22nd August 2023) through a self‐administered questionnaire using ODK software on tablets, stored on a secure server. We defined menstrual pain through two questions—“Did you have any pain during your last menstrual period?” and “What happened during your last menstrual period”. Participants were included as having menstrual pain if they reported yes to ‘any pain’ or reported a symptom of headache, backache, stomach ache or cramp at LMP.

The outcomes for this paper, assessed at trial endline, were (i) use of at least one pain management strategy defined a priori as “effective” (i.e., painkillers, stretching, eating water‐rich foods, exercising, drinking water, warm water bottle on the stomach) and none of the “ineffective” strategies (taking antibiotics, eating spicy foods, drinking soda) at LMP, among those who reported menstrual pain at LMP; (ii) perceived menstrual pain relief among those using any pain management strategy at LMP (through the question “How much of the pain did this reduce overall during your period?”); (iii) school absence (reported number of days of missing school due to menstruation in the past two terms [February 6th to May 5th and May 29th to August 22nd 2023]) and (iv) educational performance through an examination set and marked by the Uganda National Examination Board.

The exposures were baseline measures of individual‐level (age, student type, religion, ethnicity, meals eaten the previous day as a proxy of socio‐economic status), household‐level (socio‐economic status, household size, caregiver education, primary caregiver) and school‐level characteristics (school ownership, school category, district); and endline measures of (i) social support during menstruation; (ii) mental health problems measured using the Strengths and Difficulties Questionnaire Total Difficulties Score (SDQ); (iii) knowledge of puberty and menstruation (number of 9 knowledge items answered correctly; Table S1), attitudes towards menstruation (number of 3 items answered positively; Table S1), and knowledge of at least one pain management strategy defined a priori as effective (“What are good ways to manage pain?”); (iv) the Menstrual Practice Needs Scale (MPNS) [20] (extent to which menstrual management practices and environments were perceived to meet an individuals' needs during their LMP) with a mean score 0 (more unmet needs) to 3 (fewer unmet needs); and (v) the Self‐Efficacy in Addressing Menstrual Needs Scale (SAMNS) [21] (participants' confidence in their capabilities to address their menstrual needs) with scores from 0 (poor self‐efficacy) to 100 (high self‐efficacy). The SAMNS includes 3 sub‐scales, assessing self‐efficacy in menstrual hygiene preparation and maintenance, menstrual pain management and execution of stigmatised tasks respectively.

#### 2.2.2 Qualitative Data

Focus group discussions (FGDs) and in‐depth interviews (IDIs) were conducted by experienced researchers in four purposively‐selected schools in the intervention arm [19] using a semi‐structured guide. These explored participants' experiences of pain, pain management strategies, pain relief and school absenteeism. We conducted 8 FGDs and 12 IDIs with adolescent girls (half immediately after intervention delivery and half at endline). Interviews and discussions were conducted in English or Luganda, depending on participants' preference.

### 2.3 Analysis Methods

**FIGURE 1:**
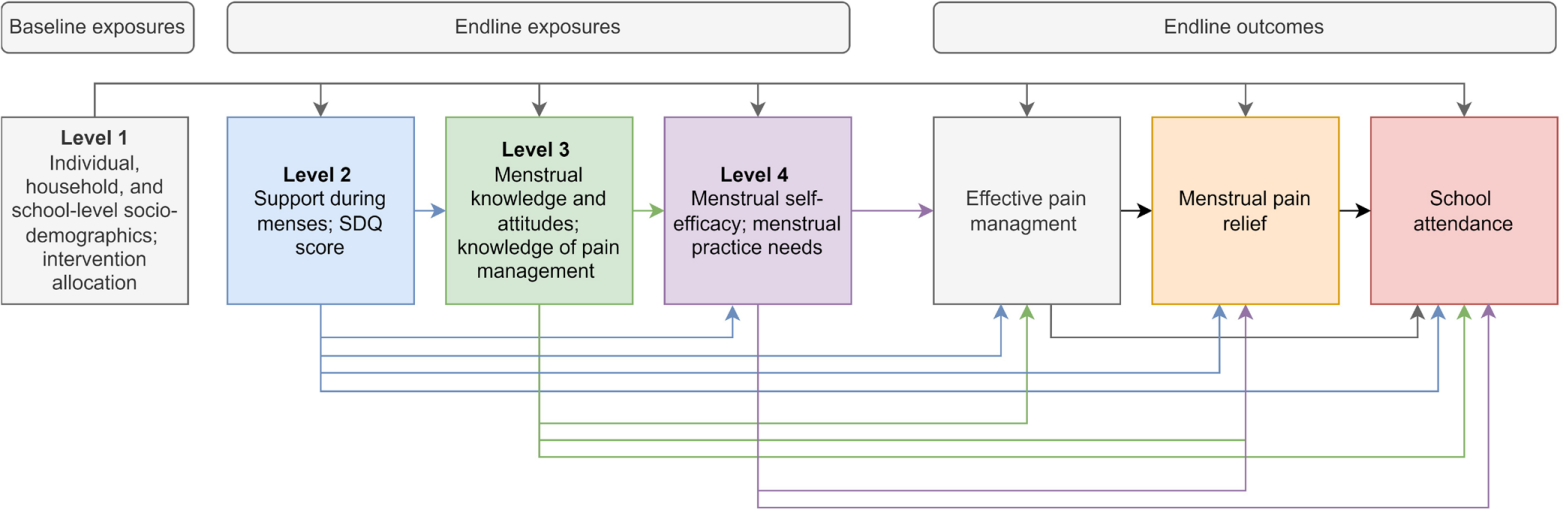
Conceptual hierarchical framework.

Statistical analyses were conducted using Stata version 18.0. We used logistic regression models to estimate the adjusted odds ratios (aOR) and 95% confidence intervals (CI) for associations between exposures and reported use of an effective pain management strategy at LMP and reported pain relief respectively, adjusting for school‐level clustering with random effects and intervention arm (as a fixed‐effect a priori defined confounding variable due to associations with the outcome and multiple exposures of interest in this paper [18]). We fitted multivariable models by adjusting each variable for others on the same and more distal levels (Figure 1). We used negative binomial regression models to estimate (i) the rate of days of school absenteeism overall and due to menstruation per month; and (ii) the adjusted incidence rate ratios (aIRR) and 95% CI for the association of these outcomes with menstrual pain, use of an effective pain management strategy, pain relief and number of menstrual symptoms. We used mixed‐effects linear regression for the analogous analysis of examination performance at endline.

Qualitative interviews were audio‐recorded, transcribed verbatim, and translated into English. Transcripts were reviewed for completeness and accuracy before coding. A thematic framework was chosen to allow structured exploration of pre‐identified issues (guided by the intervention theory of change) and flexibility to capture unanticipated insights [22]. The coding framework was developed deductively from the interview guide. Inductive codes were generated to capture issues not anticipated a priori. Coding was conducted systematically and refined through iterative team discussions. Codes were grouped and developed into themes capturing experiences of menstrual pain, approaches to pain relief, and associations of pain management with school absenteeism and educational performance. Themes were refined for coherence and consistency, and illustrative quotations were selected to demonstrate key findings. Discrepancies in coding or theme development were discussed within the research team until consensus was reached.

## 3 Results

Of 3841 female participants enrolled at baseline, 2901 (75.5%) were seen at endline (median follow‐up: 13.1 months, range: 11.4–16.6 months). At endline, 2683 (92.5%) participants reported a menstrual period in the past 6 months and are included in the analysis population. Most participants (71.4%) were Christian, 45.8% were boarding students, 58.6% had their mother as the primary caregiver and 17.5% reported having one or fewer meals the previous day. The mean age was 16.5 years (SD 0.9) at endline.

### 3.1 Prevalence of Menstrual Pain and Use of Pain Management Strategies at Endline

Overall, 2227 (83.0%) participants reported any menstrual pain at LMP at endline. Of the 1973 (88.6%) reporting a specific symptom, 1818 (92.1%) reported stomach pain, back pain or cramps.

**FIGURE 2:**
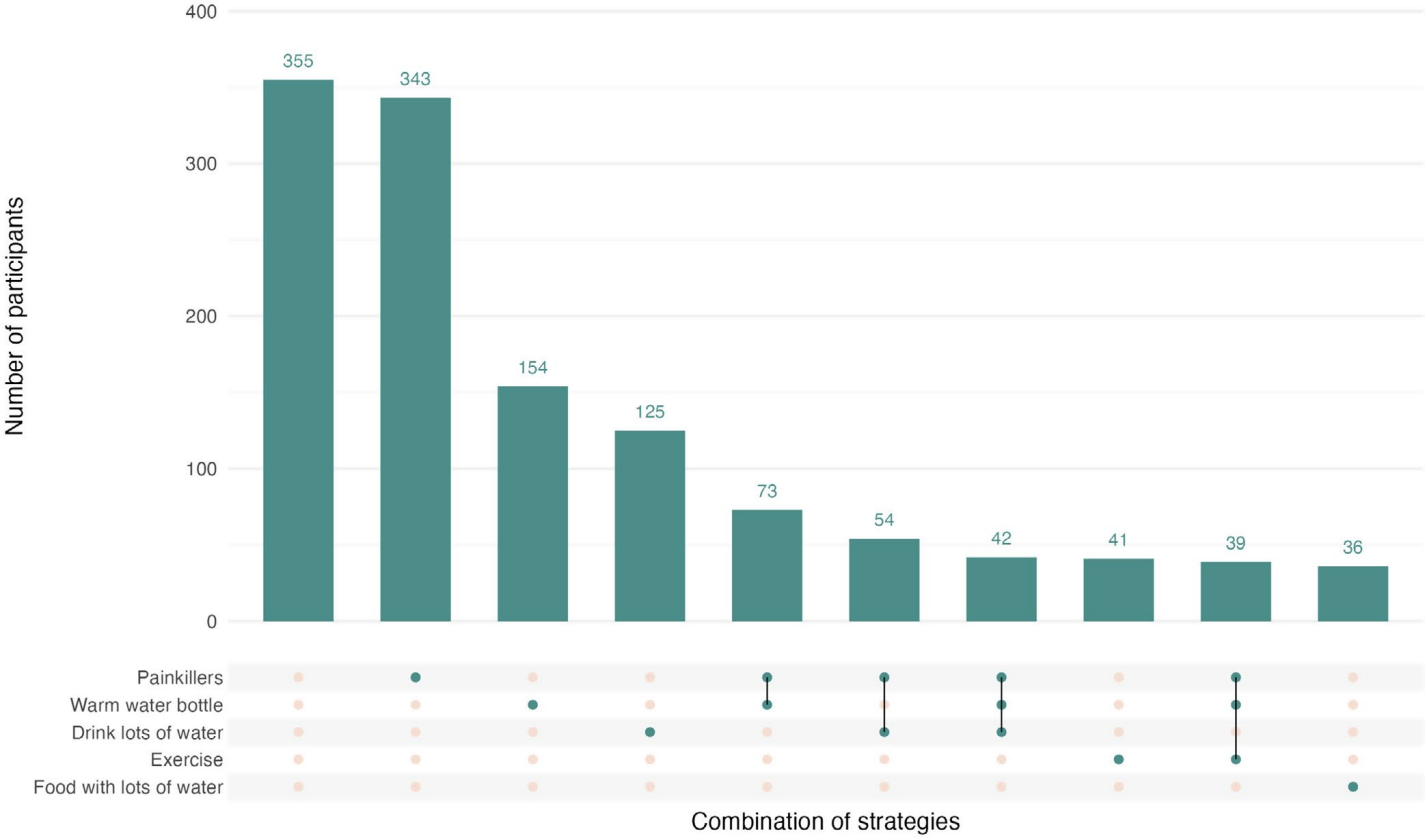
Frequency of pain management strategies.

Almost all participants (*n* = 2623; 97.9%) reported knowledge of at least one effective pain management strategy, and 1587/2226 (71.3%) of those with pain at LMP reported using an effective pain management strategy at LMP and none of the ineffective strategies listed (data on pain management was missing for one participant reporting pain). Of these, about half (*n* = 813; 51.2%) reported relief of all or most menstrual pain. The most common pain management strategies were the use of painkillers (*n* = 343, 15.4%), warm water bottle use (6.9%) or drinking lots of water (5.6%) (Table 1). Painkillers were commonly used in combination with other strategies (Table 1; Figure 2). Overall, 355 (15.9%) of those with pain at LMP reported doing nothing to ease the pain.

**TABLE 1:** Pain management methods used at last menstrual period (LMP) at endline, overall and by arm, among 2226 participants reporting pain at LMP.

Pain relief method reported at LMP	*N* (%)	Control arm (*n* = 1141)		Intervention arm (*n* = 1085)	
		*N* (%)	All or most of pain relieved, *n* (%)	*N* (%)	All or most of pain relieved, *n* (%)
Any method defined as ‘effective’	1587 (71.3)	761 (48.0)	361 (47.4)	826 (52.1)	452 (54.7)
**Single strategies**					
Painkillers only	343 (15.4)	156 (45.5)	85 (54.5)	187 (54.5)	111 (60.0)
Warm water bottle use alone	154 (6.9)	84 (54.6)	31 (36.9)	70 (45.5)	28 (40.0)
Drinking lots of water alone	125 (5.6)	77 (61.6)	34 (44.2)	48 (38.4)	11 (22.9)
Exercise only	41 (1.9)	21 (51.2)	10 (47.6)	20 (48.8)	8 (40.0)
Food with lots of water only	36 (1.6)	24 (66.7)	8 (33.3)	12 (33.3)	10 (83.3)
**Combined strategies**					
Painkillers and warm water bottle use	73 (3.3)	31 (42.5)	20 (64.5)	42 (57.5)	23 (54.8)
Painkillers and drinking lots of water	54 (2.4)	31 (57.4)	14 (45.2)	23 (42.6)	15 (65.2)
Painkillers, drinking lots of water and warm water bottle use	42 (1.9)	16 (38.1)	9 (56.3)	26 (61.9)	16 (61.5)
Painkillers, warm water bottle use and exercise	39 (1.8)	10 (25.6)	4 (40.0)	29 (74.4)	19 (65.5)
Pain killers, warm water bottle use, exercise, drinking lots of water, stretching and eating food with lots of water	31 (1.4)	9 (29.0)	3 (33.3)	22 (71.0)	17 (77.3)
Pain killers, drinking lots of water and eating food with lots of water	31 (1.4)	17 (54.8)	5 (29.4)	14 (45.2)	10 (71.4)
Pain killers, drinking lots of water and exercise	30 (1.4)	15 (50.0)	9 (60.0)	15 (50.0)	10 (66.7)

*Note:* Data on pain management at LMP was missing for one of the 2227 participants in the study.

### 3.2 Factors Associated With Use of Effective Pain Management Strategies and Pain Relief

Few socio‐demographic factors were associated with the use of effective pain management or pain relief (Table S2). The use of effective pain management was less common among participants with poorer menstrual knowledge (aOR = 0.64, 95% CI 0.42–0.99 for low vs. high knowledge scores), poorer knowledge of pain management (aOR = 0.23, 95% CI 0.13–0.42), and poorer menstrual self‐efficacy (aOR = 0.62, 95% CI 0.48–0.81) (Table 2). Among the 1871 participants who used at least one pain management strategy (including those defined a priori as ineffective), 953 (50.9%) reported relief of all or most of the pain (“pain relief”). Pain relief was less common among those with no social support for menstruation (aOR = 0.75, 95% CI 0.57–1.00), poorer knowledge of menstruation (aOR = 0.60, 95% CI 0.37–0.97), more unmet menstrual practice needs (aOR = 0.76, 95% CI 0.57–1.00), and poorer menstrual self‐efficacy (aOR = 0.66, 95% CI 0.50–0.87) (Table 2).

**TABLE 2:** Association of menstrual factors with use of effective pain management and perceived pain relief.

Variables	Use of at least one effective pain management strategy^a^ (*N* = 2226)			All/most pain relieved pain management users (*N* = 1871)	
	Frequency (%)	Yes	Fully adjusted OR (95% CI)^b^	Yes	Fully adjusted OR (95% CI)^b^
**Level 3: Menstrual related factors**					
Knowledge of menstruation			*p* trend = 0.05		*p* trend = 0.01
High (7–9)	749 (33.6)	562 (75.0)	1	359 (55.9)	1
Medium (4–6)	1370 (61.6)	957 (69.9)	0.87 (0.70, 1.07)	560 (49.0)	0.81 (0.66, 1.00)
Low (0–3)	107 (4.8)	68 (63.6)	0.64 (0.42, 0.99)	34 (39.5)	0.60 (0.37, 0.97)
Attitudes of menstruation			*p* = 0.49		*p* = 0.80
High 2–3	1716 (77.1)	1250 (72.8)	1	759 (52.0)	1
Low 0–1	510 (22.9)	337 (66.1)	0.92 (0.74, 1.15)	194 (47.3)	0.97 (0.77, 1.22)
Knowledge of pain management			*p* < 0.001		*p* trend = 0.46
Two or more	1485 (66.7)	1067 (71.9)	1	672 (52.0)	1
One	695 (31.2)	505 (72.7)	1.18 (0.96, 1.45)	271 (49.1)	0.96 (0.78, 1.19)
None	46 (2.1)	15 (32.6)	0.23 (0.13, 0.42)	10 (37.0)	0.64 (0.29, 1.43)
**Level 4: Menstrual experiences**					
Menstrual practice needs score (MPNS)			*p* = 0.28		*p*‐trend = 0.002
Few unmet needs (2.375–3)	1094 (49.2)	810 (74.0)	1	519 (56.3)	1
Some unmet needs (1.875–2.375)	632 (28.4)	433 (68.5)	0.89 (0.71, 1.13)	254 (48.3)	0.84 (0.66, 1.06)
Many unmet needs (0.00–1.875)	497 (22.4)	342 (68.8)	1.10 (0.84, 1.46)	179 (42.5)	0.76 (0.57, 1.00)
Menstrual self‐efficacy (SAMNS)			*p*‐trend < 0.001		*p*‐trend = 0.04
High (76.15–100)	740 (33.3)	571 (77.2)	1	385 (58.2)	1
Medium (59.23–75.77)	756 (33.9)	543 (71.8)	0.66 (0.53, 0.82)	338 (53.0)	0.79 (0.63, 0.98)
Low (0–58.85)	730 (32.8)	473 (64.1)	0.62 (0.48, 0.81)	230 (40.3)	0.66 (0.50, 0.87)
Menstrual Preparedness SAMNS sub‐scale^c^			*p*‐trend = 0.06		*p* = 0.04
High (76.2–100)	719 (32.3)		1	358 (57.3)	1
Medium (59.2–75.77)	763 (34.3)		0.99 (0.56, 0.93)	316 (49.9)	0.97 (0.76, 1.25)
Low (0–58.85)	744 (33.4)	510 (68.6)	1.34 (0.98, 1.82)	279 (45.5)	1.36 (0.99, 1.86)
Completing Stigmatising Tasks SAMNS sub‐scale^c^			*p*‐trend = 0.32		*p* = 0.28
High (76.2–100)	686 (30.8)	535 (67.8)	1	340 (56.1)	1
Medium (59.2–75.77)	751 (33.7)	531 (70.7)	0.90 (0.70, 1.16)	329 (51.6)	1.00 (0.78, 1.29)
Low (0–58.85)	789 (35.4)	521 (76.0)	0.87 (0.67, 1.14)	284 (45.3)	0.84 (0.64, 1.10)
Pain Management SAMNS sub‐scale^c^			*p*‐trend < 0.001		*p* trend < 0.001
High (76.2–100)	733 (32.9)	580 (79.1)	1	414 (61.5)	1
Medium (59.2–75.77)	781 (35.1)	575 (73.6)	0.72 (0.56, 0.93)	348 (51.8)	0.69 (0.55, 0.87)
Low (0–58.85)	712 (32.0)	432 (60.7)	0.42 (0.32, 0.55)	191 (36.3)	0.37 (0.28, 0.49)

^a^ Painkillers, use of warm water bottle, drinking lots of water, exercising, stretching and eating food with lots of water.

^b^ Adjusted for variables at the same or more distal levels and intervention arm.

^c^ Menstrual self‐efficacy sub‐scales are adjusted for each other and variables at the same or more distal levels, but not for overall menstrual self‐efficacy.

### 3.3 Experiences of Menstrual Pain and Pain Relief

In qualitative interviews, some participants reported menstrual pain that severely impacted their activities of daily living. Pain management strategies used were influenced by the severity and duration of pain, symptom type, convenience and availability of management methods, and personal beliefs and attitudes. Some participants preferred painkillers to other methods because they provided rapid pain relief and were easily accessible through trained school nurses in intervention schools.

Painkillers are quick at calming down the pain, they are also easy to access… So, if you feel cramps, it is easy for you to quickly get painkillers and swallow to calm down the pain. IDI Female student, Kalungu Endline

Students reported that using non‐pharmaceutical strategies alleviated pain and increased confidence in pain management.

They helped me because I used to think that to reduce pain, I take tablets but now I know I can take warm water … and life goes on FGD Female students, Kalungu Endline

Others preferred strategies we had defined as ineffective, which they perceived as working more quickly than painkillers:

If you use herbal, it may take about 5 minutes for you to feel relief. However, painkillers take some hours to work yet you are in intense pain; it can take about 30 minutes. But with herbal, it may take about 5 minutes for you to feel the change. IDI Female student, Wakiso Midline

Students reported using multiple pain management methods to address different symptoms.

I drink a lot of water, and I can also use a bottle with warm water which I put on the stomach to help relieve the pain. For the headache I get painkillers from the nurse using my vouchers. IDI Female student, Wakiso Endline

Many participants highlighted the importance of social support from family, teachers and peers, especially trusted sources of information (e.g., senior women teachers). Some reported receiving misinformation on pain management, including about potential side effects of painkillers (risk of cancer, infertility or addiction), and were advised to tolerate the pain as a natural part of being a woman.

Some people say that it is not safe to use painkillers while in their periods because it can bring cancer, but when we received the puberty lessons the teachers said other tablets can have serious side effects but then paracetamol and ibuprofen have no problem. FGD Female students, Wakiso Endline

They used to tell me not to take the medicine that it's not good. People at home used to tell me that no matter how much pain you get, just bear, be a woman, don't try to be weak. FGD Female students, Wakiso Midline

Participants reported that education sessions delivered as part of the intervention improved menstrual pain management knowledge and attitudes, dispelling misconceptions and increasing confidence to try new options.

They told me that if you take painkillers due to menstrual related pain then your chances to bear children reduce and that scared me, but ever since we were educated, I take the painkillers. FGD Female students, Wakiso Midline

### 3.4 Association of Menstrual Pain and Pain Management With School Absenteeism and Educational Performance

Among the 2682 participants with retrospective data on school absenteeism, the mean number of self‐reported days missed per month for any reason was 2.02 (95% CI 1.85–2.19), and the number missed due to menstruation was 0.30 per month (95% CI 0.27–0.34). Participants with pain at LMP were more likely to miss at least one school day per month due to menstruation than those without pain at LMP (12.0% vs. 3.3%). After adjusting for potential confounders (Levels 1–4; Figure 1), participants with pain at LMP missed more days of school overall (mean 2.1 vs. 1.4 per month; aIRR = 1.46, 95% CI 1.30–1.65; Table 3) compared to those with no pain at LMP, and missed almost 3 times as many school days during menstruation (mean 0.4 vs. 0.1 per month; aIRR = 2.89, 95% CI 2.08–4.01). There was no evidence of an association of school absenteeism with the use of effective pain management strategies (Table 3). However, students reporting none or some of their pain relieved were more likely to miss school during menstruation than those with all or most of their pain relieved (mean days missed during menstruation 0.5 vs. 0.3; aIRR = 1.39, 95% CI 1.11, 1.74) (Table 3).

**TABLE 3:** Association of school absenteeism with experience of menstrual pain, pain relief and number of symptoms.

	*N*	Mean days missed overall per month	Adjusted IRR (95% CI)^a^	*p*	Mean days missed during menstruation per month	Adjusted IRR (95% CI)^a^	*p*
**Experience of menstrual pain**							
No	456	1.4 (1.2, 1.6)	1	< 0.001	0.1 (0.07, 0.14)	1	< 0.001
Yes	2226	2.1 (2.0, 2.3)	1.46 (1.30, 1.65)		0.4 (0.3, 0.4)	2.89 (2.08–4.01)	
**Use of at least one effective pain management strategy (among those with pain at LMP)**							
Yes	1586	2.1 (1.9, 2.3)	1	*p* = 0.77	0.4 (0.3, 0.4)	1	*p* = 0.09
No	639	2.2 (2.0, 2.4)	1.02 (0.92, 1.13)		0.3 (0.3, 0.4)	0.81 (0.64, 1.03)	
**Pain relief (among those who used at least one strategy at LMP)**							
All or most of pain relieved	952	2.1 (1.9, 2.4)	1	0.73	0.3 (0.2, 0.4)	1	0.004
None/minimal pain relieved	918	2.2 (2.0, 2.4)	0.98 (0.87, 1.10)		0.5 (0.4, 0.5)	1.39 (1.11, 1.74)	

^a^ Adjusted for school clustering, intervention arm, randomisation variables and potential confounders at Levels 1–4 in Figure 1.

There was little quantitative evidence that either pain or pain management was associated with educational performance (Table 4).

**TABLE 4:** Association of educational performance with experience of menstrual pain, pain relief and number of symptoms.

	*N*	Mean UNEB score	Minimally adjusted mean difference (95% CI)^a^	*p*	Fully adjusted mean difference (95% CI)^b^	*p*
**Experience of menstrual pain**						
No	419	0.13 (0.05, 0.21)	1	0.11	1	0.49
Yes	2047	0.01 (−0.03, 0.04)	−0.06 (−0.14, 0.01)		−0.03 (−0.10, 0.05)	
**Use of at least one effective pain management strategy (among those with pain at LMP)**						
Yes	1450	0.01 (−0.03, 0.05)	1	*p* = 0.60	1	*p* = 0.72
No	596	0.00 (−0.06, 0.07)	−0.02 (−0.09, 0.05)		0.02 (−0.05, 0.08)	
**Pain relief (among those who used at least one strategy at LMP)**						
All or most of pain relieved	876	0.02 (−0.03, 0.07)	1	0.08	1	0.33
None/minimal pain relieved	835	−0.05 (−0.10, 0.01)	−0.07 (−0.14, 0.01)		−0.04 (−0.11, 0.04)	

^a^ Adjusted for school clustering, intervention arm, randomisation variables and inflated for missing school for any reason.

^b^ Adjusted for school clustering, intervention arm, randomisation variables, potential confounders at Levels 1–4 in Figure 1.

In qualitative interviews, participants reported that pain hindered their ability to focus and engage in learning activities, contributing to absenteeism and reduced class participation. Other students reported attending classes but that their menstrual pain led to decreased motivation, reduced focus, and poorer engagement in class lessons.

My menstrual cycle affects my moods, making me feel disconnected and unenthusiastic about certain subjects, and even the teachers can't engage me on those days. I attend classes, but my mind is elsewhere, and I struggle to absorb the lesson, feeling completely unmotivated and disinterested. IDI‐FEMALE students‐Wakiso Midline

After receiving the intervention, students reported reduced menstrual pain, and improved engagement in class.

…we used to have students suffering from pain when in their menstruation, they would wish to abstain from class, keeping at the [sickbay] and fail to come for classes, but nowadays they feel comfortable to attend. After the pain management training, you find that there's a big reduction of students failing to attend school. FGD‐Female students Kalungu‐Endline

## 4 Discussion

### 4.1 Main Findings

This study adds to the sparse literature on menstrual pain in Africa, and confirms the high prevalence seen in studies from Nigeria, Ethiopia and Ghana [6, 11]. Menstrual pain was prevalent among secondary school students in Uganda and was associated with increased school absenteeism but not educational performance. Participants with poorer mental health, menstrual knowledge and menstrual self‐efficacy were less likely to use effective pain management, and less likely to report pain relief. Participants reporting no or little pain relief and more menstrual pain symptoms were more likely to miss school due to menstruation than those with pain relief or fewer symptoms, respectively.

### 4.2 Strengths and Limitations

Strengths include the use of validated measures of multiple dimensions of menstrual health, self‐completion of surveys on tablets (minimising observer bias), and the high response rate and few exclusion criteria (minimising selection bias). The study is larger than those in the systematic review on PD and academic impact in young women [11], and is the only quantitative study on this topic from eastern Africa, to our knowledge. The sample of 60 secondary schools from peri‐urban and rural settings may enhance the generalisability of findings within Uganda and in settings with comparable cultural norms and educational structures. However, given the school‐based setting, the results may not be generalisable to all young women in Uganda.

As we analysed cross‐sectional data at endline, we could not assess the direction of causality for associations. Pain management would not plausibly impact knowledge and attitudes, but could improve self‐efficacy. The relationship between pain and poor mental health is likely to be bi‐directional [23], and the observed association that students with poorer mental health had poorer pain management and pain relief may be partly due to reverse causality. Similarly, the association between pain and school absenteeism may reflect some reverse causality if participants missing school were less likely to receive education about pain management. There may also be residual confounding by unmeasured factors including pain severity and type of dysmenorrhea. Further limitations were the lack of data on pain severity, or on menstrual phase, and use of self‐reported retrospective data on school absenteeism. The self‐reported absenteeism data may underestimate true absenteeism [24], but the bias is likely to be non‐differential with respect to menstrual pain or pain relief, which would underestimate the true association. Further our findings of the association of pain and lack of pain relief are supported by the qualitative findings, and in line with the literature [1].

### 4.3 Interpretation

Menstrual pain causes physical, social, and economic challenges that may impede students' ability to attend or fully participate in school, resulting in poorer academic performance [25]. We found strong evidence that menstrual pain is associated with school absenteeism, and that perceived pain relief was associated with less school absence due to menstruation, although the number of days missed due to menstruation is small (0.3 per month). Our findings support the quantitative systematic review [1] which found that menstrual pain is associated with school absenteeism, and additionally estimates the number of school days missed. The lack of evidence of an association between the use of effective pain management and school absenteeism may be because these students had more severe pain than those who did not use these strategies. Our qualitative findings support the quantitative literature that menstrual pain can affect academic performance both through school absenteeism and poorer engagement in class among students who attended school but were in pain [1].

Students reported that menstrual pain negatively affected their mood, and that participants with poor mental health were less likely to use effective pain management strategies, which aligns with other studies [26]. Longitudinal studies are needed to clarify the directionality and mechanisms underlying this association, including evaluation of interventions that address both menstrual pain and psychological distress [26].

Participants who used effective pain management strategies tended to have better general menstrual knowledge and self‐efficacy. This may reflect knowledge influencing menstrual self‐efficacy leading to better menstrual effective pain management. This is consistent with a hypothesis from previous qualitative studies in LMICs that capabilities influence confidence [21]. Those with confidence in their ability to manage their pain may be more likely to seek support for pain management.

As expected, the use of effective pain management was associated with knowledge of pain management strategies. This, along with the intervention effect on effective pain management in the MENISCUS trial [18], highlights the need for improved education about pain management strategies, especially in contexts where there are common misconceptions about painkillers.

## 5 Conclusions

Effective menstrual pain management is associated with other dimensions of menstrual health and with mental health. Pain relief can be enhanced through targeted menstrual health interventions that focus on increasing knowledge and menstrual confidence, social support and access to pain relief strategies. Menstrual pain is associated with increased school absenteeism, which can negatively impact educational outcomes. Future research is needed to refine menstrual health interventions to better support pain management and menstrual stigma.

To effectively implement such interventions, collaboration across stakeholders, including governments, non‐governmental organisations, and school communities will be essential in addressing the misconceptions around menstrual pain management strategies. These collective efforts have the potential to contribute to reducing school absenteeism, and improving school engagement and overall well‐being for adolescents.

## Author Contributions

Conceptualisation: H.A.W., B.T.‐L., P.N., B.N., R.K., A.N., C.B., S.B., D.S., D.N., K.A.N.; Methodology: H.A.W., B.T.‐L., K.A.N.; Statistical analysis: C.B., H.A.W.; Investigation: P.N., A.N., C.B., B.N., R.K., S.B., D.S., E.M.H., S.N., K.A.T.,D.N., K.A.N.; Writing – original draft: P.N., A.N., R.K., S.B.,D.N., B.T.‐L., H.A.W.; Writing – review and editing: All authors; Supervision and funding acquisition: H.A.W.

## Funding

This work was funded by the Joint Global Health Trials Scheme with funding from the UK Foreign, Commonwealth and Development Office (FCDO), the UK Medical Research Council (MRC), the UK Department of Health and Social Care (DHSC) through the National Institute for Health Research (NIHR), and the Wellcome Trust (Grant Ref. MR/V005634/1) awarded to HAW. The funders had no role in conducting research, analysis or drafting manuscripts.

## Ethics Statement

Ethics approval for the study was granted by the Uganda Virus Research Institute Research & Ethics Committee (May 26, 2021; GC/127/21/05/819), the Uganda National Council of Science and Technology (July 14, 2021; HS1525ES) and the London School of Hygiene & Tropical Medicine Interventional Research Ethics Committee (August 3, 2021; 22952). We sought consent from parents of participants under 18 years old, and from participants aged 18 years and above. Participants aged less than 18 years assented before participating in the study. The trial is registered as ISRCTN 45461276.

## Conflicts of Interest

The authors declare no conflicts of interest.

## Supporting information


**Table S1:** MENISCUS knowledge and attitude questions about puberty and menstruation.
**Table S2:** Associations of socio‐demographic factors, social support and mental health with use of effective pain management and perceived pain relief.

## Data Availability

Data are available upon request from https://datacompass.lshtm.ac.uk/id/eprint/3822/.
