# Supplementary material for: Menstrual Pain Management, School Absenteeism and Educational Performance Among Adolescent Students: Cross‐Sectional Mixed‐Methods Analysis Nested Within a Cluster Randomised Trial
Source: BJOG. 2025 Nov 24;133(4):761–70. doi: 10.1111/1471-0528.70094 (PMC12884233; doi:10.1111/1471-0528.70094)
Supplement: Supplementary file 1 — Table S1: MENISCUS knowledge and attitude questions about puberty and menstruation. Table S2: Associations of socio‐demographic factors, social support and mental health with use of effective pain management and perceived pain relief. [file BJO-133-761-s001.docx]

**Table S1: MENISCUS knowledge and attitude questions about puberty and menstruation**

| **Knowledge questions** | **Response options (correct response in bold)** |
| --- | --- |
| K1: What is menstrual period blood? | - Blood from the stomach - **Blood from the lining of the uterus (womb)** - Don't know |
| K2: The physical changes related to puberty usually start between 10 and 14 years of age in girls, and between 12 and 16 in boys. | - **True** - False - Don't know |
| K3: Changes in the body during puberty happen because of hormones | - **True** - False - Don't know |
| K4: How long does a menstrual period (bleeding) usually last? | - **Between 3 to 7 days** - Exactly 5 days - About 28 days - Don't know |
| K5: Monthly menstruation continues during pregnancy | - True - **False** - Don't know |
| K6: Usually, women stop menstruating after about age 40-50 years | - **True** - False - Don't know |
| K7: When during the menstrual cycle is a woman most likely to become pregnant? | - Just before her period - During her period - Right after her period - **After ovulation** - Don’t know |
| K8: What is the entrance to the uterus called? | - Vagina - Vulva - **Cervix** - Ovary - Don’t know |
| K9: How many days are there usually between menstrual periods? | - Exactly 7 days - Exactly 28 days - **Between 21-45 days** - Don’t know |
| **Attitude questions** |  |
| A1: It is fine for a girl to cook during her period | - Disagree a lot - Disagree - Neither agree nor disagree - **Agree** - **Agree a lot** |
| A2: It is fine for a girl to run, dance or cycle during her period |  |
| A3: Painkillers cause problems having children (barrenness) | - **Disagree a lot** - **Disagree** - Neither agree nor disagree - Agree - Agree a lot |

**Table S2: Associations of socio-demographic factors, social support and mental health with use of effective pain management and perceived pain relief**

| **Variables** | **Use of at least one effective pain management strategy^1^ (N=2,226)** | | | | **Reported all or most pain relieved among those who used pain management (N=1,871)** | |
| --- | --- | --- | --- | --- | --- | --- |
|  | **Frequency (%)** | | **Yes** | **Fully adjusted OR (95%CI)^2^** | **Yes** | **Fully adjusted OR (95%CI)^2^** |
| **Level 1: Socio-demographic and socio-economic** | | | | |  |  |
| **District** |  | |  | P=0.43 |  | P=0.76 |
| Wakiso | 1743 (78.3) | | 1228 (70.5) | 1 | 739 (51.0) | 1 |
| Kalungu | 483 (21.7) | | 359 (74.3) | 1.13 (0.84, 1.52) | 214 (50.6) | 1.04 (0.81,1.34) |
| **School ownership** | | |  | P=0.06 |  | P=0.06 |
| Private | 1404 (63.1) | | 970 (69.1) | 1 | 578 (49.8) | 1 |
| Government | 822 (36.9) | | 617 (75.1) | 1.30 (0.99,1.70) | 375 (52.7) | 1.24 (0.99,1.55) |
| **Schooling category** | | |  | P=0.93 |  | P=0.84 |
| Day | 1229 (55.2) | | 890 (72.4) | 1 | 534 (50.9) | 1 |
| Boarding | 997 (44.8) | | 697 (69.9) | 0.99 (0.81,1.22) | 419 (50.9) | 0.98 (0.80,1.20) |
| **Age categories (years)** | | |  | P=0.66 |  | P=0.82 |
| <15 | 189 (8.5%) | | 131 (69.3) | 1 | 85 (55.2) | 1 |
| 16 | 991 (44.5%) | | 702 (70.8) | 1.01 (0.72,1.41) | 415 (51.0) | 0.87 (0.61, 1.23) |
| 17 | 792 (35.6%) | | 576 (72.7) | 1.05 (0.74,1.49) | 344 (49.9) | 0.85 (0.59, 1.22) |
| >18 | 254 (11.4%) | | 178 (70.1) | 0.86 (0.56,1.31) | 109 (51.2) | 0.91 (0.59, 1.41) |
| **Ethnicity** |  | |  | P=0.57 |  | P=0.34 |
| Muganda | 1546 (69.5) | | 1113 (72.0) | 1 | 676 (51.4) | 1 |
| Non Muganda | 680 (30.6) | | 474 (69.7) | 0.94 (0.77,1.16) | 277 (49.8) | 0.90 (0.73,1.11) |
| **Religion** |  | |  | P=0.26 |  | P=0.65 |
| Catholic | 740 (33.3) | | 539 (72.8) | 1 | 311 (49.6) | 1 |
| Other Christian | 837 (37.6) | | 582 (69.5) | 0.84 (0.67,1.16) | 359 (52.3) | 1.14 (0.91, 1.42) |
| Muslim | 643 (28.9) | | 461 (71.7) | 1.00 (0.77,1.28) | 281 (50.7) | 1.05 (0.82, 1.33) |
| None/Other | 6 (0.3) | | 5 (83.3) | 2.81 (0.33,24.0) | 2 (40.0) | 0.68 (0.11, 4.23) |
| **Primary caregiver** | | |  | P=0.71 |  | P=0.16 |
| Mother | 1295 (58.2) | | 933 (72.0) | 1 | 562 (51.6) | 1 |
| Father | 550 (24.7) | | 385 (70.0) | 0.91 (0.73,1.14) | 220 (47.6) | 0.83 (0.66,1.03) |
| Other | 381 (17.1) | | 269 (70.6) | 0.97 (0.75,1.25) | 171 (53.4) | 1.06 (0.82,1.38) |
| **Social Economic Status (SES)** | | |  | P=0.21 |  | P-trend=0.02 |
| Lowest | 441 (19.8) | | 324 (73.5) | 1 | 179 (46.9) | 1 |
| Medium-low | 462 (20.8) | | 321 (69.5) | 0.92 (0.69,1.23) | 183 (47.9) | 1.03 (0.77,1.37) |
| Medium | 429 (19.3) | | 317 (73.9) | 1.21 (0.89,1.65) | 197 (53.4) | 1.25 (0.93, 1.69) |
| Medium-high | 467 (21.0) | | 323 (69.2) | 0.99 (0.73,1.34) | 197 (50.9) | 1.17 (0.87, 1.59) |
| Highest | 427 (19.2) | | 302 (70.7) | 1.24 (0.90,1.47) | 197 (56.1) | 1.44 (1.04, 2.00) |
| **Meals eaten on the previous day** | | | | P=0.66 |  | P-trend=0.25 |
| Three or more | 709 (31.9) | | 494 (69.7) | 1 |  | 1 |
| Two | 1128 (50.7) | | 813 (72.1) | 1.09 (0.88, 1.34) |  | 0.91 (0.73, 1.12) |
| One or less | 389 (17.5) | | 280 (72.0) | 1.11 (0.84, 1.47) |  | 0.85 (0.65, 1.13) |
| **Level 2: Social support during menstruation & mental health problems** | | | | | |  |
| **Social support** |  | |  | P=0.19 |  | P=0.05 |
| Yes | 1927 (86.6) | | 1385 (71.9) | 1 | 846 (52.0) | 1 |
| No | 299 (13.4) | | 202 (67.6) | 0.84 (0.64, 1.08) | 107 (43.9) | 0.75 (0.57, 1.00) |
| **Mental health problems (SDQ score)** | | | | P=0.03 |  | P=trend=0.007 |
| Few problems (0-8) | | 820 (36.8) | 607 (74.0) | 1 | 372 (54.3) | 1 |
| Medium (9-13) | 766 (34.7) | | 550 (71.8) | 0.87 (0.70, 1.08) | 340 (52.3) | 0.93 (0.74, 1.16) |
| Many problems (14-40) | 640 (28.8) | | 430 (67.2) | 0.73 (0.58, 0.92) | 241 (45.0) | 0.71 (0.56, 0.90) |

^1^ Effective pain management strategies are painkillers, use of warm water bottle, drinking lots of water, exercising, stretching and eating food with lots of water

^2^ Adjusted for variables at the same or more distal levels. Variables at Levels 2-4 are adjusted for intervention arm.
